# I_Ks_ Activator ML277 Mildly Affects Repolarization and Arrhythmic Outcome in the CAVB Dog Model

**DOI:** 10.3390/biomedicines11041147

**Published:** 2023-04-11

**Authors:** Joanne J. A. van Bavel, Henriëtte D. M. Beekman, Agnieszka Smoczyńska, Marcel A. G. van der Heyden, Marc A. Vos

**Affiliations:** Department of Medical Physiology, Division of Heart & Lungs, University Medical Center Utrecht, 3584 Utrecht, The Netherlands

**Keywords:** ML277, I_Ks_ channel, AV block dog model, long QT type 1, ventricular arrhythmia

## Abstract

Long QT syndrome type 1 with affected I_Ks_ is associated with a high risk for developing Torsade de Pointes (TdP) arrhythmias and eventually sudden cardiac death. Therefore, it is of high interest to explore drugs that target I_Ks_ as antiarrhythmics. We examined the antiarrhythmic effect of I_Ks_ channel activator ML277 in the chronic atrioventricular block (CAVB) dog model. TdP arrhythmia sensitivity was tested in anesthetized mongrel dogs (n = 7) with CAVB in series: (1) induction experiment at 4 ± 2 weeks CAVB: TdP arrhythmias were induced with our standardized protocol using dofetilide (0.025 mg/kg), and (2) prevention experiment at 10 ± 2 weeks CAVB: the antiarrhythmic effect of ML277 (0.6–1.0 mg/kg) was tested by infusion for 5 min preceding dofetilide. ML277: (1) temporarily prevented repolarization prolongation induced by dofetilide (QTc: 538 ± 65 ms at induction vs. 393 ± 18 ms at prevention, *p* < 0.05), (2) delayed the occurrence of the first arrhythmic event upon dofetilide (from 129 ± 28 s to 180 ± 51 s, *p* < 0.05), and (3) decreased the arrhythmic outcome with a significant reduction in the number of TdP arrhythmias, TdP score, arrhythmia score and total arrhythmic events (from 669 ± 132 to 401 ± 228, *p* < 0.05). I_Ks_ channel activation by ML277 temporarily suppressed QT interval prolongation, delayed the occurrence of the first arrhythmic event and reduced the arrhythmic outcome in the CAVB dog model.

## 1. Introduction

Long QT syndrome (LQTS) can be either congenital or acquired and is characterized by a prolongation of the QT interval on the electrocardiogram (ECG). When inherited, the most common type is caused by a loss-of-function mutation in the *KCNQ1* gene, referred to as LQT type 1 (LQT1), and is found in 40–50% of LQTS individuals [1,2]. The *KCNQ1* gene encodes the α-subunit of the slow component of the delayed rectifier potassium current (I_Ks_) and forms a functional channel together with the β-subunit (KCNE1). Specific activation of the channel is present upon enhanced sympathetic stimulation and when maintaining a proper repolarization reserve [3,4]. A loss-of-function mutation in the *KCNQ1* gene results in a reduced I_Ks_ density [5]. The accompanying reduced repolarization reserve predisposes the heart to Torsade de Pointes (TdP) ventricular arrhythmias and possibly sudden cardiac death. Enhanced sympathetic activity, typically by physical exercise or emotional stress, is reported as a trigger [6].

Insights into regulators of KCNQ1/KCNE1 channels have been reported over the last decades. They range from physiological modulators protein kinase A, phosphatidylinositol 4,5-biphosphate and adenosine triphosphate to pharmacological modulators of which, among others, hexachlorophene, zinc pyrithione and L-364,373 are presented as I_Ks_ activators [7,8,9,10]. The latter are critically discussed regarding their sensitivity of solely KCNQ1 or the KCNQ1/KCNE1 complex and their effect on other (cardiac) ion channels in terms of possible side effects for LQT1 patients.

(R)-N-(4-(4-methoxyphenyl)thiazol-2-yl)-1-tosylpiperidine-2-carboxamide (ML277) was identified as a novel I_Ks_ activator by Mattmann and coworkers in 2012 [11]. It has been proposed that activation of the I_Ks_ channel is achieved by increasing K^+^ conductance and by prolonging the activation and deactivation transitions, and even prevention of channel inactivation, by selectively enhancing the current during the activated open state of the channel [12,13,14]. Furthermore, ML277 showed a very modest or no effect on the L-type calcium current (I_CaL_) and the inward-rectifying current (I_K1_) in guinea pig ventricular cardiomyocytes [12]. Since its identification, a few in vitro studies have reported on the therapeutic potential of ML277. By acting on KCNQ1 and KCNQ1/KCNE1 complexes, ML277 enhances I_Ks_ density and shortens the action potential duration in canine ventricular cardiomyocytes and human-induced pluripotent stem-cell-derived cardiomyocytes (hiPSC-CMs) [12,15]. ML277 rescued I_Ks_ dysfunction in hiPSC-CMs with a patient-specific *KCNQ1* mutation as shown via elevated I_Ks_ density and action potential shortening [16]. Similar results by ML277 were shown in patient-specific hiPSC-CM clusters based on a reduced field potential duration [17].

To our knowledge, the potential antiarrhythmic efficacy of ML227 in vivo has not yet been reported. The chronic atrioventricular block (CAVB) dog is a model in which TdP arrhythmias can be induced in serial experiments with high reproducibility, and has been widely used to explore pro- and antiarrhythmic drug effects [18]. Ventricular remodeling after AV block in combination with bradycardia and anesthesia predispose the heart to TdP arrhythmias, and the I_Kr_ blocker dofetilide is accountable as a final trigger for TdP induction [19,20]. The aim of this study is to examine the potential antiarrhythmic effect of the I_Ks_ activator ML277 in the CAVB dog with a focus on repolarization duration and arrhythmic outcome.

## 2. Materials and Methods

### 2.1. Animals

Animal care and experimentation were approved by the Committee for Experiments on Animals of Utrecht University and were in accordance with the Directive 2010/63/EU of the European Parliament and the Dutch law on animal experimentation (application approval number: AVD115002016531, date of approval: 6 August 2016). Dogs were housed in pairs in kennels with wooden bedding material, had ad libitum access to drinking water and received food pellets twice a day. The animals were allowed to play outside once a day with access to playing toys, and their welfare was checked daily.

The experiments were performed with seven purpose-bred mongrel dogs (two females) (Marshall, New York, NY, USA). All dogs had remodeled hearts caused by AV block, which was induced by radiofrequency ablation of the His bundle [21]. The animals had a body weight of 25 ± 3 kg and were 18 ± 2 months old.

### 2.2. Preparation

Animals were fasted overnight and received premedication (0.02 mg/kg i.m. atropine, 0.5 mg/kg i.m. methadone, 0.5 mg/kg i.m. acepromazine and 0.1 mg/kg s.c. meloxicam) half an hour prior to the procedure. General anesthesia was induced by sodium pentobarbital (Nembutal, 25 mg/kg i.v.) and maintained by 1.5% isoflurane in O_2_ and N_2_O (1:2 ratio) via mechanical ventilation at 12 breaths/min. Ampicillin (1000 mg) was administered before (i.v.) and after (i.m.) surgery, and buprenorphine (0.3 mg, i.m.) was provided after surgery. A monophasic action potential (MAP) catheter (Hugo Sachs Elektronik, March, Germany) was inserted via the jugular artery to the left ventricular apex. Surface ECG and a MAP signal were recorded continuously during the experiment using EP Tracer (Cardiotek, Maastricht, The Netherlands) with a sampling rate of 1 kHz. Five dogs had an idioventricular rhythm (IVR), whereas two dogs were paced at VVI40-50 due to extreme bradycardia at IVR. The induction and prevention experiments were performed in series.

### 2.3. Compounds

I_Kr_ blocker dofetilide (Biorbyt, Cambridge, United Kingdom, 0.025 mg/kg, i.v.) was dissolved in 0.1 M HCl and further prepared in 0.9% saline solution. Infusion time was 5 min or until occurrence of the first TdP arrhythmia. I_Ks_ activator ML277 (Bio-Connect B.V., Huissen, The Netherlands, 0.6–1 mg/kg, i.v.) was dissolved in polyethylene glycol 400 (PEG 400) and dimethyl sulfoxide (DMSO) in a 1:1 ratio. The dose was based on absence of electrophysiological and hemodynamic effects upon proof-of-principle cardiac safety experiments in anesthetized sinus rhythm dogs.

### 2.4. Induction Experiment

**Figure 1:**
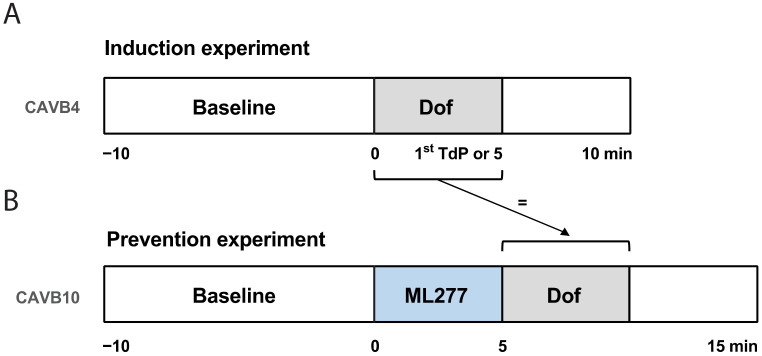
Schematic overview of the experimental setup. Two experiments were performed in series. (**A**) Induction experiment at chronic AV block 4 ± 2 weeks (CAVB4): baseline measurement followed by dofetilide (Dof) infusion until first Torsade de Pointes (TdP) arrhythmia or maximum infusion of 5 min. (**B**) Prevention experiment at CAVB 10 ± 2 weeks (CAVB10): baseline measurement followed by 5 min of ML277 and Dof infusion with identical duration as during the induction experiment. Recordings were followed up for 10 min after the start of Dof administration.

TdP arrhythmias were induced via a standardized protocol. After 10 min of baseline (BL) recording, I_Kr_ blocker dofetilide was infused for 5 min or until the occurrence of the first TdP (Figure 1A). TdP arrhythmias that lasted longer than 10 s were terminated via defibrillation. Dogs were considered inducible when they showed at least three TdP arrhythmias (of ≥5 ectopic beats) within 10 min after the start of dofetilide infusion. Seven inducible dogs at 4 ± 2 weeks of CAVB remodeling (CAVB4) were included for the prevention experiment.

### 2.5. Prevention Experiment

At 10 ± 2 weeks of CAVB remodeling (CAVB10), the antiarrhythmic potential of I_Ks_ activator ML277 was examined in inducible dogs. After 10 min of BL recording, ML277 was infused for 5 min followed by dofetilide with a similar infusion duration as the induction experiment (Figure 1B). Within the same animal, the dofetilide infusion time in the prevention experiment was exactly the same as the dofetilide infusion time during the induction experiment (until the first arrhythmic event or 5 min). This timepoint is referred to as dofetilide timepoint 1 (Dof T1).

### 2.6. Data Analysis

Five consecutive beats from ECG lead II were measured manually in EP Tracer to obtain the interval duration of ECG parameters RR, PP, QRS and QT. The QT interval was corrected for heart rate (QTc) using the Van de Water formula [22]. The JTc interval was obtained by subtracting the QRS interval from the QTc interval. The MAP duration of the left ventricle (LV MAPD) at 80% of repolarization was measured semi-automatically using custom-made software (AutoMAPD, MATLAB, MathWorks, Natick, MA, USA). The beat-to-beat variability of repolarization, quantified as short-term variability (STV), was calculated from 31 consecutive beats using the formula: $STV=\sum \left|D_{n+1}-D_{n}\right|/\left(30*\sqrt{2}\right)$, with D representing the LV MAPD [23].

Arrhythmic events were scored to quantify the severity of the arrhythmic outcome [24]. Single ectopic beats (sEB) were scored with 2 points, multiple ectopic beats (mEB) were scored with 3–5 points and self-terminating TdP arrhythmias were scored with 6–49 points. TdP arrhythmias received a score of 50, 75 or 100 points for one, two or three defibrillations, respectively. The arrhythmic outcome was quantified by the relative TdP score (scored TdP arrhythmias relative to the induction experiment), the total number of shocked TdP arrhythmias, and the total score of all arrhythmic events within the 10 min time window after the start of dofetilide infusion. The arrhythmia score is based on the average of the three highest scored arrhythmic events [24].

### 2.7. Statistical Analysis

Data are presented as mean ± standard deviation (SD). Serial data were analyzed using a paired Student’s *t*-test or a one-way analysis of variance (ANOVA) with a Tukey’s test to correct for multiple comparisons. All statistical analyses were performed with GraphPad Prism (version 8.3.0, GraphPad Software, San Diego, CA, USA). A value of *p* < 0.05 was considered statistically significant.

## 3. Results

### 3.1. Temporary Suppression of Repolarization Prolongation

**Figure 2:**
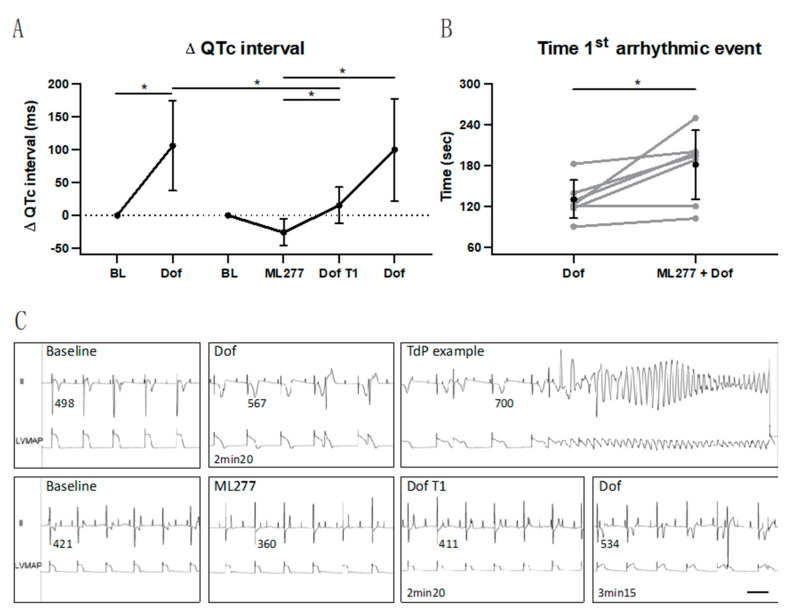
Effect of ML277 in dogs (n = 7) with chronic AV block (CAVB). (**A**) Progress of the delta QTc interval (in ms) in the induction experiment: chronic AV block (CAVB) 4 weeks at baseline (BL) and before the first arrhythmic event during dofetilide (Dof). The prevention experiment at CAVB10 included timepoints at BL, at 5 min of ML277 infusion (ML277), at the same timepoint as the first arrhythmic event during Dof at CAVB4 (Dof T1) and at the actual first arrhythmic event during Dof. Data of individual dogs in grey and mean ± SD in black, repeated measures one-way ANOVA with Tukey’s multiple comparisons test, * *p* < 0.05. (**B**) Time until the first arrhythmic event occurred during Dof at CAVB4 and during Dof after ML277 at CAVB10. Paired *t*-test, * *p* < 0.05. (**C**) Representative ECG lead II and left ventricular monophasic action potential (LV MAP) tracings (dog #7) with QT intervals (in ms) presented at each measured timepoint and before the shocked Torsade de Pointes (TdP) arrhythmia (example during Dof at CAVB4). The scale bar is 1000 ms.

The duration of dofetilide infusion—established during the induction experiment—was identical to the dofetilide duration at the prevention experiment (172 ± 54 s, corresponding to 57 ± 18% of a full dose at 5 min), referred to as Dof T1 timepoint. An overview of the delta QTc interval progress upon the induction and prevention experiments is presented in Figure 2A. During the induction experiment, dofetilide significantly prolonged the QTc interval. During the prevention experiment, ML277 shortened the QTc interval to some extent in all dogs. Moreover, the QTc interval prolongation during dofetilide was suppressed by the I_Ks_ activator as shown by a shorter QTc interval at Dof T1 (393 ± 18 ms) compared to this timepoint during dofetilide of the induction experiment (538 ± 65 ms, *p* < 0.05, Figure 2A and Table 1).

However, this was a temporary effect since dofetilide further prolonged the QTc interval—measured at the timepoint before the actual first arrhythmic event upon dofetilide at the prevention experiment (479 ± 82 ms, Figure 2A and Table 1). Repolarization parameters QT, JTc and LV MAPD showed the same behavior as the QTc interval: prolongation upon dofetilide, a trend towards shortening upon ML277 and a delay upon dofetilide following ML277 at Dof T1 (Table 1). The progress of the QT interval at the different timepoints is also represented in the ECG lead II tracings of one dog in Figure 2C. The RR interval and QRS duration remained stable at all timepoints (Table 1). The atrial rate was not affected by ML277, whereas dofetilide showed an increased PP interval compared to the baseline in the prevention experiment. The delayed effect by ML277 is also found in the temporal dispersion of repolarization parameter STV: a dofetilide duration, similarly to the induction experiment (Dof T1), did not increase the STV yet (0.85 ± 0.41, Table 1). ML277 infusion alone did not induce any arrhythmic events.

### 3.2. Delay in Occurrence of First Arrhythmic Event

The timepoint at which the first arrhythmic event occurred after the onset of dofetilide infusion is presented in Figure 2B. The time interval at which the first arrhythmic event occurred upon dofetilide following ML277 pretreatment was significantly longer (180 ± 51 s) compared to the time at which the first arrhythmic event occurred upon solely dofetilide infusion (129 ± 28 s, *p* < 0.05).

### 3.3. Mild Antiarrhythmic Effect: Occurrence of TdP Arrhythmias

**Figure 3:**
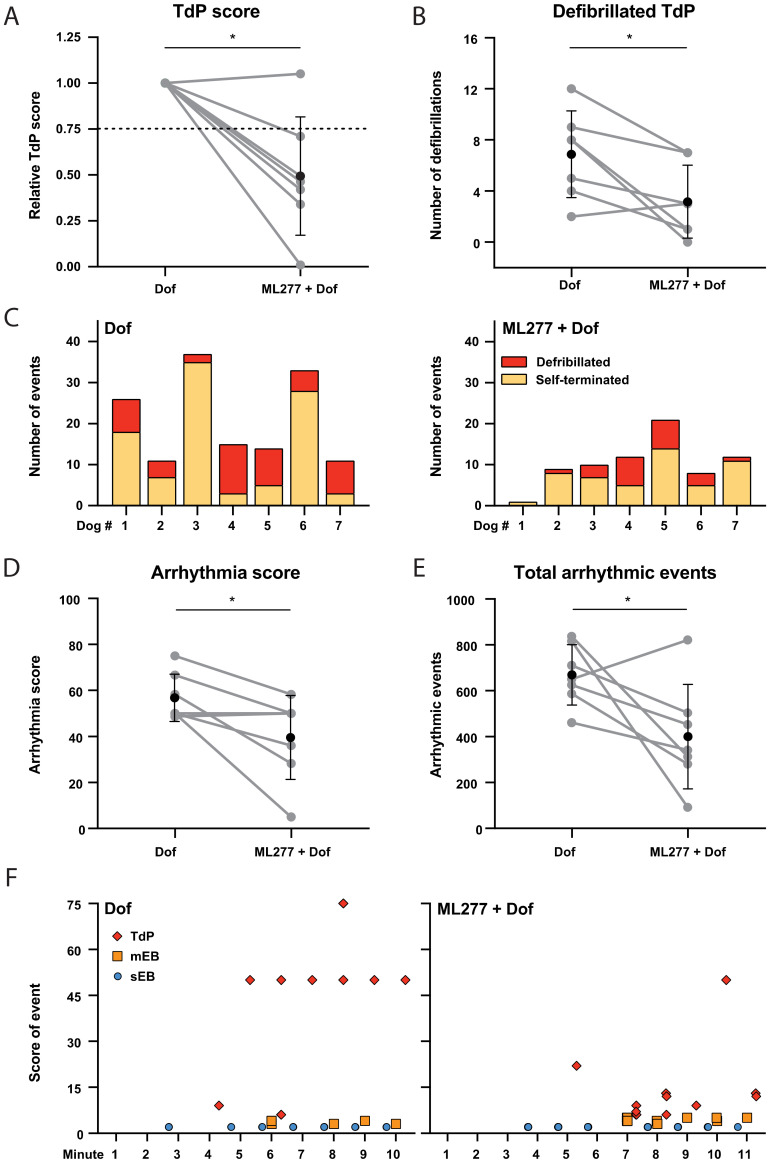
Reduction in Torsade de Pointes (TdP) arrhythmias after ML277. (**A**) Relative TdP score and (**B**) number of TdP arrhythmias terminated by defibrillation during dofetilide (Dof) infusion and during Dof following ML277. (**C**) Overview of defibrillated and self-terminated TdP arrhythmias per dog during Dof and ML277 + Dof. (**D**) Arrhythmia score and (**E**) total of arrhythmic events during Dof and ML277 + Dof. Data of individual dogs in grey and mean ± SD in black, paired *t*-test with * *p* < 0.05. (**F**) Representative overview of arrhythmic events in the 10 min time window per minute after the start of Dof (left panel) and after Dof following ML277 (right panel). The score of arrhythmic events refers to single ectopic beats (sEB, 2 points), multiple ectopic beats (mEB, 3–5 points) and TdP arrhythmias (TdP, 6–75 points). A TdP terminated with one shock was scored with 50 points, and two defibrillations were scored with 75 points. A delay of 55 s of the 10 min time window is included in the results of ML277 + Dof. Results are from dog #7.

I_Ks_ activation by ML277 did not solely delay the occurrence of the first arrhythmic event: the infusion of ML277 before the induction of TdP arrhythmias by dofetilide reduced the relative TdP score in six out of seven dogs by more than 25% (Figure 3A). The number of TdP arrhythmias terminated by defibrillation was also significantly lower following ML277 pretreatment (Figure 3B). The number of defibrillated vs. self-terminated TdP arrhythmias per dog are presented in Figure 3C and show the overall reduction in the arrhythmias after ML277.

The reduced arrhythmic outcome by ML277 is also presented by the significant decrease in the arrhythmia score (Figure 3D) and the total score of arrhythmic events (Figure 3E). The 10 min time window after the onset of dofetilide infusion, in which the arrhythmic events were quantified, was shifted at the prevention experiment to compensate for the delayed occurrence of the first arrhythmic event. A representative overview of the occurrence of arrhythmic events during the prevention and induction experiment is presented in Figure 3F. Note the delay of the first arrhythmic event with ML277 (140 s at Dof vs. 195 s at ML277 + Dof) and the reduced number of defibrillated TdP arrhythmias with a score equal to or higher than 50.

## 4. Discussion

In this study, we examined the potential antiarrhythmic effects of I_Ks_ activator ML277 in the CAVB dog model, which allows reproducible inducement of TdP arrhythmias under standardized conditions. ML277 temporarily suppressed dofetilide-induced repolarization prolongation and delayed the occurrence of the first arrhythmic event. Furthermore, the arrhythmic outcome was reduced as quantified by the TdP score, the number of defibrillated TdP arrhythmias, the arrhythmia score, and the total score of arrhythmic events.

### 4.1. Targeting I_Ks_ in the CAVB Dog Model

The I_Kr_ and I_Ks_ are crucial in accomplishing a stable repolarization phase, where I_Ks_ is less dominant compared to I_Kr_ [25]. Furthermore, both currents strongly contribute to a safety mechanism in case of an impaired function of a single potassium channel. The contribution of I_Ks_ to this repolarization reserve is more dominant: I_Kr_ blocking by dofetilide prolonged the APD by approximately 30%, while around 50% of the prolongation was due to the HMR-1556-induced loss of I_Ks_ in dog cardiomyocytes [25]. In the CAVB dog model, the I_Kr_ and I_Ks_ densities are significantly downregulated [26]. This explains the ML277-induced delay in repolarization prolongation upon dofetilide in the CAVB dog, and a lack of ML277-induced QTc modulation in sinus rhythm dogs. I_Ks_ blocking by JNJ303 prolonged the QT interval in the CAVB model, and in combination with a trigger as enhanced inotropy, ventricular tachycardia and TdP arrhythmias were induced [27].

In our standardized induction protocol, it is likely that I_Kr_ blocker dofetilide sufficiently prolongs the QT interval in such a way that the remaining I_Ks_ fails to compensate to maintain a proper repolarization reserve on a cellular level. This, in combination with AV block-induced contractile and structural remodeling of the ventricles, anesthesia and bradycardia, predisposes the heart to TdP arrhythmias in approximately 75% of the animals [20]. The current study included solely animals inducible for TdP arrhythmias upon dofetilide infusion. ML277 activated the I_Ks_ remaining after AV block-induced downregulation and the inhibiting effect of anesthesia component isoflurane [28]. This is presented by a delay in the occurrence of the first arrhythmic event and a delay in QT interval prolongation. Then, dofetilide challenged the repolarization reserve to such an extent to become reduced and unstable, thereby inducing QT prolongation and arrhythmic events. Here, the prevention approach (ML277 before dofetilide) instead of a suppression experiment (ML277 after dofetilide) allowed the establishment of electrophysiological effects of ML277 itself, and a controlled measurement of electrophysiological parameters such as STV [29], which would be challenging to obtain upon enhanced arrhythmogenicity when dofetilide was given first. For further investigation, a suppressive approach would be of clinical value in determining a therapeutic effect.

### 4.2. Reproducibility of Dofetilide-Induced Arrhythmias

With I_Kr_ blocker dofetilide as the final hit in the standardized protocol of the CAVB dog model, TdP arrhythmia can be reproducibly induced in serial experiments [30]. Here, an average number of 13 TdP arrhythmias occurred in the 10 min dofetilide time window, of which 20% demanded defibrillation. In the current study, inducible animals ranged highly in the number of defibrillations upon dofetilide (2–12). Upon ML277, a significant reduction in the arrhythmic outcome was presented, though the occurrence of TdP arrhythmias—including the severe events demanding defibrillation—could not be suppressed completely. Here, the results of the induction and prevention experiments were compared in order to examine the potential antiarrhythmic outcome of ML277. While the exact reproducibility of the arrhythmic outcome during two serial experiments cannot be excluded, the TdP outcome remained relatively stable between the serial experiments upon a similar dose of dofetilide [30].

In terms of cardiac remodeling over time, TdP sensitivity in inducible animals is consistent over the weeks—from CAVB2 weeks on [20]. Moreover, KCNQ1 mRNA and protein levels already decreased at three days, were maintained at for least 30 days after the AV block in serial experiments [31]. An essential component for creating vulnerable circumstances for TdP arrhythmias to occur in our CAVB dog model is anesthesia induced by pentobarbital and maintained by isoflurane. Important to note is that no arrhythmias occur under baseline conditions with this anesthetic regime [32]. On a level of ionic currents, isoflurane has a blocking effect on I_Ks_ [28]. The effect of isoflurane in the current study can be excluded due to serial performance of the induction and prevention experiment under identical conditions.

### 4.3. (Pre)Clinical Implications

Despite the reduced repolarization prolongation and arrhythmic outcome, the sustained occurrence of TdP arrhythmias after ML277 upon dofetilide infusion fails to consider this I_Ks_ activator as an antiarrhythmic drug in our preclinical model [33]. Yet, this study is the first to determine I_Ks_ activating effects of the compound in an animal model with bradycardia-induced sensitivity to ventricular arrhythmias. Furthermore, it reveals a potential role of I_Ks_ activation in long QT circumstances (temporal antiarrhythmic effect) across the well-determined and more dominant aspect of I_Kr_.

### 4.4. Study Limitations

Despite the extensive electrophysiological evaluation of ML277 in the CAVB dog model, the current data cannot distinguish if dofetilide counteracts with or ‘overrules’ the ML277-induced electrophysiological effects or if it concerns a potential limited duration of action by ML277. I_Ks_ and action potential recordings are lacking in this study and could have provided further insight into the time and dose-dependent behavior of ML277 with a confirming evaluation of the presented in vivo electrophysiological effects of ML277. Additional pharmacokinetic analysis is required to elaborate on the peak plasma concentrations and its corresponding time-dependent effect on the presented antiarrhythmic properties.

## 5. Conclusions

I_Ks_ activation by ML277 temporarily suppresses dofetilide-induced repolarization prolongation and the occurrence of a first arrhythmic event in the CAVB dog model. Whereas the arrhythmic outcome is significantly reduced upon ML277, it fails to completely suppress dofetilide-induced TdP arrhythmias.

## Figures and Tables

**Table 1:** Electrophysiological parameters from seven chronic AV block (CAVB) dogs after dofetilide (Dof), and after ML277 followed by Dof.

	Induction		Prevention			
	BL	Dof	BL	ML277	Dof T1	Dof
**RR**	1275 ± 190	1285 ± 180	1322 ± 242	1399 ± 299	1398 ± 301	1432 ± 290
**PP**	583 ± 113	638 ± 101	577 ± 101	596 ± 114	611 ± 102	672 ± 143 *
**QRS**	104 ± 21	102 ± 27	107 ± 24	107 ± 25	104 ± 27	103 ± 29
**QT**	456 ± 93	563 ± 70 *	406 ± 40	387 ± 36	428 ± 27 ^#^	516 ± 77 *^&%^
**QTc**	432 ± 84	538 ± 65 *	378 ± 26	352 ± 31	393 ± 18 ^#^	479 ± 82 *^&%^
**JTc**	327 ± 74	436 ± 47 *	272 ± 40	245 ± 34	289 ± 19 ^#^	376 ± 59 *^&%^
**LV MAPD**	308 ± 41	399 ± 77 *	278 ± 20	274 ± 26	315 ± 24 ^#^	389 ± 53 *^&%^
**STV**	1.63 ± 0.50	2.97 ± 0.91	0.78 ± 0.56	0.73 ± 0.33	0.85 ± 0.41	2.31 ± 1.44 *^&^

Parameters in milliseconds, data presented as mean ± SD, at baseline (BL) and before the first arrhythmic event during Dof at the induction experiment at CAVB 4 weeks, and at 5 min of ML277 infusion (ML277), at the same timepoint as the first arrhythmic event during Dof at CAVB 4 weeks (Dof T1), and at the actual first arrhythmic event during Dof at the prevention experiment at CAVB 10 weeks. LV MAPD = left ventricular monophasic action potential duration, STV = short-term variability of LV MAPD. N = 6 for LV MAPD and STV. Repeated measures one-way ANOVA with Tukey’s multiple comparisons test. * *p* < 0.05 compared to BL within CAVB group, ^#^ *p* < 0.05 compared to Dof CAVB4, ^&^ *p* < 0.05 compared to ML277 and ^%^ *p* < 0.05 compared to Dof T1.

## Data Availability

The data presented in this study are available on request from the corresponding author.
